# An Optimized Spline-Based Registration of a 3D CT to a Set of C-Arm Images

**DOI:** 10.1155/IJBI/2006/47197

**Published:** 2006-04-06

**Authors:** S. Jonić, P. Thévenaz, G. Zheng, L. -P. Nolte, M. Unser

**Affiliations:** ^1^ Biomedical Imaging Group, École Polytechnique Fédérale de Lausanne (EPFL), Lausanne VD CH-1015, Switzerland; ^2^ Institut de Minéralogie et de Physique des Milieux Condensés, Université Pierre et Marie Curie, Paris 75015, France; ^3^ Institute for Surgical Technology and Biomechanics, MEM Research Center for Orthopaedic Surgery, University of Bern, Bern CH-3014, Switzerland

## Abstract

We have developed an algorithm for the rigid-body registration of a CT volume to a set of C-arm images. The algorithm uses a gradient-based iterative minimization of a least-squares measure of dissimilarity between the C-arm images and projections of the CT volume. To compute projections, we use a novel method for fast integration of the volume along rays. To improve robustness and speed, we take advantage of a coarse-to-fine processing of the volume/image pyramids. To compute the projections of the volume, the gradient of the dissimilarity measure, and the multiresolution data pyramids, we use a continuous image/volume model based on cubic B-splines, which ensures a high interpolation accuracy and a gradient of the dissimilarity measure that is well defined everywhere. We show the performance of our algorithm on a human spine phantom, where the true alignment is determined using a set of fiducial markers.


Hindawi Publishing Corporation
International Journal of Biomedical Imaging
Volume 2006, Article ID 47197, Pages 1–12
DOI 10.1155/IJBI/2006/47197

### An Optimized Spline-Based Registration of a 3D CT to a Set of C-Arm Images

**S. Jonić,[1,2] P. Thévenaz,[1] G. Zheng,[3] L.-P. Nolte,[3] and M. Unser[1]**

[1] *Biomedical Imaging Group, École Polytechnique Fédérale de Lausanne (EPFL), CH-1015 Lausanne VD, Switzerland*
[2] *Institut de Minéralogie et de Physique des Milieux Condensés, Université Pierre et Marie Curie, 75015 Paris, France*
[3] *Institute for Surgical Technology and Biomechanics, MEM Research Center for Orthopaedic Surgery, University of Bern, CH-3014, Bern, Switzerland*

Received 12 October 2005; Revised 23 January 2006; Accepted 3 February 2006

Recommended for Publication by Tiange Zhuang

We have developed an algorithm for the rigid-body registration of a CT volume to a set of C-arm images. The algorithm uses a gradient-based iterative minimization of a least-squares measure of dissimilarity between the C-arm images and projections of the CT volume. To compute projections, we use a novel method for fast integration of the volume along rays. To improve robustness and speed, we take advantage of a coarse-to-fine processing of the volume/image pyramids. To compute the projections of the volume, the gradient of the dissimilarity measure, and the multiresolution data pyramids, we use a continuous image/volume model based on cubic B-splines, which ensures a high interpolation accuracy and a gradient of the dissimilarity measure that is well defined everywhere. We show the performance of our algorithm on a human spine phantom, where the true alignment is determined using a set of fiducial markers.

Copyright © 2006 S. Jonić et al. This is an open access article distributed under the Creative Commons Attribution License, which permits unrestricted use, distribution, and reproduction in any medium, provided the original work is properly cited.

### 1. INTRODUCTION

Image-guided navigation procedures are critical components of currently available systems for computer-assisted therapy and surgery [1–4]. Orthopedic procedures offer a real-time display of tools superimposed on the three-dimensional (3D) computed tomography (CT) data that have been acquired preoperatively and that are used for planning of the surgery. CT-based navigation has been successful for spinal pedicle screw insertion, total hip arthroplasty, total knee arthroplasty, pelvic osteotomy, and reconstruction of knee cruciate ligaments [4, 5]; however, it is difficult to implement because it requires that the CT volume be registered to the patient at the time of the surgical intervention. This is the problem that we address in this paper.

A partial solution is to use fluoroscopy-based navigation, which is a recent technique that shows promising results in early clinical trials of spinal pedicle screw insertion, distal locking of femoral nails, and femoral fracture reduction [5, 6]. This technique displays the tools in a set of intraoperative images of the patient, but is limited to two-dimensional (2D) data. Images at different viewing angles are acquired by a C-arm device which has become part of the standard equipment for orthopedic surgery thanks to its mobility and ease of manipulation. To maintain the patient and the C-arm image intensifier in common registration, a position sensor tracks them simultaneously [6, 7]. The C-arm images with overlaid projections of the tools are displayed on the computer screen. An update rate of 10 Hz enables real-time navigation in up to four C-arm images simultaneously [7]. The lack of three-dimensionality can be compensated by an augmented number of image shots, which has the drawback of excessive irradiation of the surgical staff and of the patient.

There are two groups of algorithms that can register a 3D CT to a set of X-ray images [8–16]. One group matches specific anatomical features that appear in the two imaging modalities [9–12, 14, 15, 17]. These methods rely on an accurate data segmentation or edge detection. They are therefore less robust to the partial-data problem (presence of a feature in one imaging modality and its absence in the other) than projection-based (or intensity-based) methods that form the other group of registration algorithms [8, 13, 16, 18–20]. In general, these methods require little or no segmentation.

They achieve the registration by matching a set of X-ray images to their simulations. The simulations are traditionally computed as projections of the CT volume obtained by summing the volume intensities along simulated X-rays through the volume. Since the projection-based methods perform the registration of the CT volume to the X-ray images using a large amount of data, their accuracy is potentially higher than that of anatomy-based methods. In this paper, we follow the projection-based approach to combine good properties of the CT-based navigation and of the fluoroscopy-based navigation. Our goal is to achieve an improved, automatic 3D CT-based navigation which would display the tools in the preoperative 3D CT based on its registration to only a few (say, five or less) intraoperative C-arm images.

The projection-based registration is commonly achieved by optimizing a measure of similarity between the X-ray images and their simulations. To perform optimization, some practitioners use Powell's multidimensional direction set method [19, 20], while others use gradient-descent-type search technique [8]. In this paper, we perform optimization by using the Levenberg-Marquardt (LM) optimizer, which is tuned to least-squares dissimilarity measures and is very efficient. Moreover, the LM approach makes possible the simultaneous optimization of the dissimilarity measure in all unknown parameters.

Penney et al. compare six similarity measures, based on fiducial markers, for registering a 3D CT to a fluoroscopy image of a spine phantom [21]. They report pattern intensity and gradient difference as being able to register accurately and robustly, even when soft tissues and interventional instruments are present in the fluoroscopy image. They find mutual information to be the least accurate of the six similarity measures. Lemieux et al. propose to register a 3D CT to two radiographic images by optimizing a cross-correlation-based cost function at the initial stage of the registration, and a gradient-based cost function at its final stage [20]. With the same goal, Brown and Boult maximize the gradient correlation between the original and the simulated radiographs [19].

The reference image and the corresponding projection of the volume have generally different intensity ranges. Brown and Boult explore the physical relationship between CT and radiograph measurements [19]. They use it to correct the radiographs such that their registration to the 3D CT is more accurate. Penney et al. design a similarity measure with a suitable scaling of the fluoroscopy image or the volume projection which normalizes their intensity ranges [21]. In this paper, we use a least-squares dissimilarity measure, where we deal with the intensity ranges by normalizing them.

The computational effort of projection-based methods increases with the data size. Several methods for a fast computation of the volume projections, based on ideas from computer graphics, have been proposed [13, 22]. The method called shear-warp factorization consists of two steps [23]. In the first step, an intermediate image is computed. The volume, resampled at a higher resolution, is projected using nearest-neighbor interpolation in-slice only. The second step relies on a linear interpolation of the intermediate image to obtain the final projection. Light fields (a technique to parameterize the set of all rays that emanate from a static scene) allow most of the computation to be performed in a preprocessing step [13, 22]. Using a light field generated from a number of volume projections, new projections can be computed by interpolating the four-dimensional space of prestored rays. To speed up the registration, Sarrut and Clippe have proposed to precompute a set of projections of the 3D CT that are evenly distributed in space and to match them to X-ray images [18]. In a simulation study, Birkfellner et al. have shown that a 2D/2D 1-DOF (degrees of freedom) registration applied in alternation with a 3D/2D 5-DOF method accelerates the registration three times without loss in accuracy [8]. Another strategy used to improve speed performs the registration iteratively using data at multiple resolutions (multiresolution pyramid) obtained usually by data blurring, using an averaging filter, followed by downsampling [20]. Sometimes, the multiresolution pyramids are built on a region of interest (ROI) extracted from the volume and the images [21]. The multiresolution strategies have been shown to improve the robustness of the registration [24, 25].

In this paper, we propose to benefit from a continuous image/volume model based on cubic B-splines for computing the projections of the volume, the gradient of the similarity measure, and the multiresolution data pyramids [26–29]. Also, we describe a novel shearing method to compute fast projections. This is a one-step approach that does not complicate the optimization procedure, contrary to the intermediate-image approach [23]. Here, we applied our algorithm on the registration of a cadaver spine CT volume with respect to the corresponding C-arm images. The ground-truth registration parameters were estimated based on the position of fiducial markers implanted on the specimen before the data acquisition. We performed two series of experiments, the first on an ROI containing a single vertebra and the second on an ROI containing three vertebrae. The latter was performed to provide an upper bound to the registration performance, although this configuration would not be possible in clinical conditions. We also study the influence of the number of C-arm images and their relative orientation to the registration accuracy. Our algorithm requires at least two input images and its accuracy can be improved either by increasing the number of input C-arm images, or by imposing a minimal angle between their planes, or by combining both strategies.

The paper is organized as follows: we describe the geometry of the problem and the fast computation of the projections in Section 2, along with the model used for the data interpolation. The cost function and its optimizer are described in Section 3. In Section 4, we show the performance of our algorithm on the available data using an objective measure of the registration accuracy. The conclusions are given in Section 5.

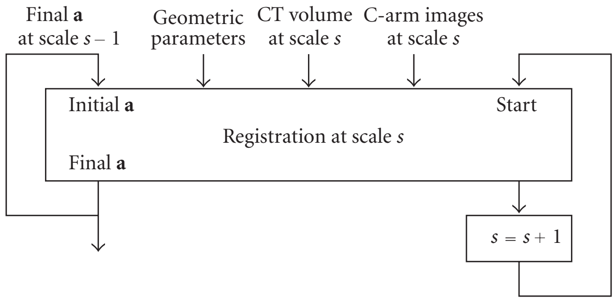

Figure 1: Multiresolution strategy.

### 2. GEOMETRICAL SETUP

### 2.1. Geometric parameters

Given a 3D CT of a patient and a set of 2D C-arm images with known positions and orientations in the reference coordinate system (R-COS), our algorithm computes a rigid-body transformation of the volume in the R-COS with respect to these images. Since we need a meaningful interpretation of the transformation in terms of clinical (anatomical) angles, we use three Euler angles (two out-of-plane rotations and one in-plane rotation) together with three translations to represent this rigid-body motion. Let therefore $\mathbf{a} = (a_1, a_2, \ldots, a_6)$ be a vector containing three Euler rotations and three translations of the volume in the R-COS. To compute these parameters, our algorithm refines their initial values by minimizing the mean-square dissimilarity between the C-arm images and the projections of the volume, which we refer to as the cost function. This corresponds to minimizing a real-valued function of the six variables $a_i$, $i = 1, 2, \ldots, 6$.

To improve the robustness and the speed of the algorithm, we register the volume to the images at different resolutions, from the coarsest resolution to the resolution that provides a good tradeoff between accuracy and time. We use the estimate of the position and of the orientation obtained at some resolution to resume the registration at the next finer one (Figure 1). To obtain data at different resolutions, we compute their B-spline least-squares $L_2$ pyramids, where $L_2$ denotes the space of measurable, square-integrable functions [30, 31].

The registration at a single resolution of the data is schematically shown in Figure 2. The input data for this registration are (1) the CT volume, (2) the C-arm images, (3) the extrinsic geometrical parameters (the voxel and pixel sizes, and, for each C-arm image, the R-COS position and orientation of the image plane and the R-COS position of the illumination source), and (4) the initial position and orientation of the volume.

### 2.2. Model

The B-spline model $f$ reconstructed from the samples $f[\mathbf{n}]$ of a function, where $\mathbf{n} = (n_1, \ldots, n_N) \in \mathbb{Z}^N$, is given by

$$f(\mathbf{x}) = \sum_{\mathbf{n}\in\mathbb{Z}^N} c[\mathbf{n}]\beta^m(\mathbf{x}-\mathbf{n}), \quad \forall \mathbf{x} = (x_1, \ldots, x_N) \in \mathbb{R}^N, \tag{1}$$

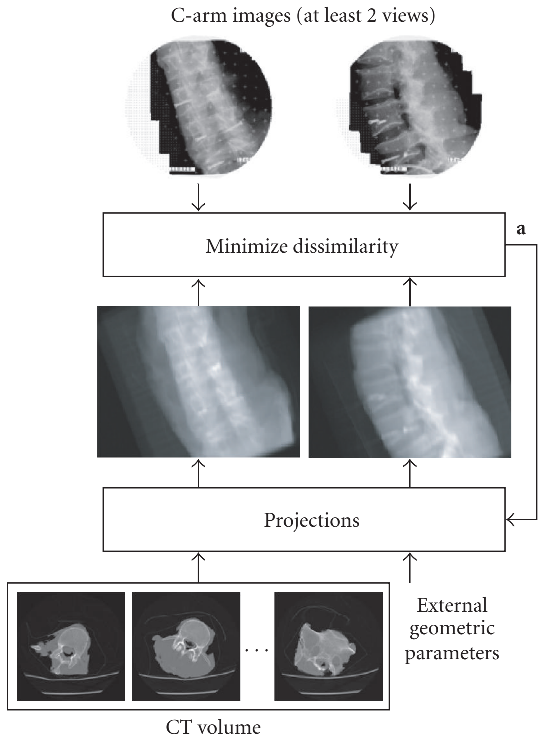

Figure 2: Algorithm for registering a 3D CT to C-arm images.

where the coefficients $c[\mathbf{n}]$ are obtained by recursive digital filtering [32] of the samples $f[\mathbf{n}]$, and where $\beta^m(\mathbf{x})$ is the separable $N$-dimensional B-spline of degree $m$ given by

$$\beta^m(\mathbf{x}) = \prod_{j=1}^{N} \beta^m(x_j). \tag{2}$$

Here, the function $\beta^m$ on the right-hand side denotes the centered one-dimensional B-spline of degree $m$ [29]. The separability makes possible an operation on $N$-dimensional data to be performed by successive processing of one-dimensional data along each of the $N$ dimensions. In return, the data processing is simple and fast.

The B-splines have many interesting properties [28]. For instance, the approximation order (and the support) of a B-spline of degree $m$ is equal to $(m + 1)$. They are shown to be maximally continuous basis functions, with the minimal support for a given order of approximation, and with the maximal order of approximation for a given support [33].

### 2.3. Projection

Let us refer to the system of 3D voxel indexes, $\mathbf{n} = (n_1, n_2, n_3, 1)$, as the volume coordinate system (V-COS) and to the system of 2D pixel indexes, $\mathbf{k} = (k_1, k_2, 0, 1)$, as the image coordinate system (I-COS). All the vectors and matrices used here are given in homogeneous coordinates. Moreover, let us define the position and the orientation of the image plane in the R-COS by the R-COS coordinate of an in-plane point (e.g., $\mathbf{p}$) and by two in-plane orthonormal vectors

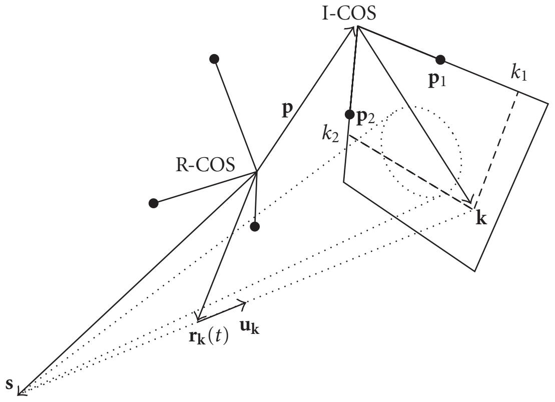

FIGURE 3: Cone-beam projection geometry.

(e.g., $\mathbf{p}_1$ and $\mathbf{p}_2$) (Figure 3). Given a volume at the position and orientation $\mathbf{a}$ in the R-COS, its B-spline model $f$, the position and the orientation of the image plane ($\mathbf{p}$, $\mathbf{p}_1$, $\mathbf{p}_2$) in the R-COS, and the R-COS coordinate of the illumination source $\mathbf{s}$, the algorithm computes a projection of the volume by casting through the model $f$ a set of simulated X-rays that meet at the source.

Although the X-rays are produced by an illumination source that has a definite physical extent, for the sake of simplicity we prefer to model it as a punctual source situated at $\mathbf{s}$. This source emits rays that hit the image plane at every pixel index $\mathbf{k}$ each ray follows the unit vector $\mathbf{u_k} = (\mathbf{A\,k}-\mathbf{s})/\|\mathbf{A\,k}-\mathbf{s}\|$, where $\mathbf{A}$ is some $(4\times 4)$ homogenous matrix that depends on the position and the orientation of the image plane in the R-COS and on the pixel size. The projection of the volume points with the R-COS coordinate $\mathbf{r_k}(t) = \mathbf{s} + t\,\mathbf{u_k}$ (i.e., along the ray determined by the unit vector $\mathbf{u_k}$) to the image plane point with the I-COS coordinate $\mathbf{k}$ is

$$p_{\mathbf{k}}(\mathbf{a}) = \int_{t_1}^{t_2} f\big((\mathbf{B}(\mathbf{a}))^{-1}\mathbf{r_k}(t)\big)\mathrm{d}t, \qquad (3)$$

where $t_1 = 0$, $t_2 = \|\mathbf{A\,k}-\mathbf{s}\|$, and where the $(4\times 4)$ matrix $\mathbf{B}(\mathbf{a})$ is the transformation from the V-COS to the R-COS. We express the transformation $\mathbf{B}(\mathbf{a})$ in Appendix A.

### 2.4. *Fast projection*

A direct implementation of (3) would require a 3D interpolation of the volume at the V-COS coordinate $(\mathbf{B}(\mathbf{a}))^{-1}\mathbf{r_k}(t)$. We perform a fast computation of the projection by replacing the 3D interpolation by a 2D interpolation. The principle of this approach is related to [34, 35]; we refer to this method as shearing.

Let us rewrite (3) as

$$p_{\mathbf{k}}(\mathbf{a}) = \int_{\mathbb{R}} f\big(\mathbf{n}_0(\mathbf{a}) + t\mathbf{n_k}(\mathbf{a})\big)\mathrm{d}t, \qquad (4)$$

where $\mathbf{n}_0(\mathbf{a}) = (\mathbf{B}(\mathbf{a}))^{-1}\mathbf{s}$ and $\mathbf{n_k}(\mathbf{a}) = (\mathbf{B}(\mathbf{a}))^{-1}\mathbf{u_k}$. The shearing method is based on a change of the integration variable $t$ in (4). The new integration variable is selected from one of three possibilities that correspond to $j \in \{1,2,3\}$, given by

$$\tau = \big[\mathbf{n}_0(\mathbf{a})\big]_j + t\big[\mathbf{n_k}(\mathbf{a})\big]_j \longrightarrow \mathrm{d}\tau = \big[\mathbf{n_k}(\mathbf{a})\big]_j\mathrm{d}t, \qquad (5)$$

where $[\mathbf{x}]_j$ is the $j$th component of the vector $\mathbf{x}$. This yields

$$p_{\mathbf{k}}(\mathbf{a}) = \lambda_{j;\mathbf{k}}(\mathbf{a}) \int_{\mathbb{R}} f\big(\mathbf{n}_{0;j;\mathbf{k}}(\mathbf{a}) + \tau\mathbf{n}_{j;\mathbf{k}}(\mathbf{a})\big)\mathrm{d}\tau, \quad j \in \{1,2,3\}, \qquad (6)$$

where $\mathbf{n}_{0;j;\mathbf{k}}(\mathbf{a}) = \mathbf{n}_0(\mathbf{a}) - ([\mathbf{n}_0(\mathbf{a})]_j/[\mathbf{n_k}(\mathbf{a})]_j)\,\mathbf{n_k}(\mathbf{a})$ and $\mathbf{n}_{j;\mathbf{k}}(\mathbf{a}) = \mathbf{n_k}(\mathbf{a})/[\mathbf{n_k}(\mathbf{a})]_j$. The scaling factor $\lambda_{j;\mathbf{k}}(\mathbf{a}) = |1/[\mathbf{n_k}(\mathbf{a})]_j|$ determines which one of the three expressions for $p_{\mathbf{k}}(\mathbf{a})$ will be used to compute the projection. We choose $j$ for which $\lambda_{j;\mathbf{k}}(\mathbf{a})$ is minimal, which ensures the highest sampling rate for $f$. Since $\lambda_{j;\mathbf{k}}(\mathbf{a})$ depends on $\mathbf{k}$, the decision about $j$ is taken independently for each pixel of the projected image.

The integral in (6) can then be approximated by a discrete sum where the size and the phase of the sampling step have been chosen so that only samples of the volume with one integer and two real coordinates take part in the sum, that is,

$$p_{\mathbf{k}}(\mathbf{a}) \approx \lambda_{\mathbf{k}}(\mathbf{a}) \sum_{n\in\mathbb{Z}} f\big(\mathbf{n}_{0;\mathbf{k}}(\mathbf{a}) + n\mathbf{n_k}(\mathbf{a})\big). \qquad (7)$$

Note that, by construction, the evaluation of $f$ requires the interpolation of the volume at a point with one integer and only two real coordinates, which saves on computations. An example of applying the shearing method for computing a 1D cone-beam projection of a 2D image is shown in Figure 4. As we can see from this figure, there are two image regions. In one (hollow circles), we sample the image along the horizontal axis, while in the other (black-filled disks), the sampling is performed along the vertical axis. (The gray samples of Figure 4 can be attributed to either region.) Likewise, we can find regions of image pixels $\mathbf{k}$ with same $j$ in the 3D-to-2D case. This can speed up the implementation of the projection equation.

By combining (1) with (7), we get the final expression that allows us to estimate the value of the projection of a volume with position and orientation $\mathbf{a}$ along a simulated X-ray of direction $\mathbf{u_k}$, onto the C-arm pixel indexed by $\mathbf{k}$, as

$$p_{\mathbf{k}}(\mathbf{a}) = \lambda_{\mathbf{k}}(\mathbf{a}) \sum_{n\in\mathbb{Z}} \sum_{\mathbf{n}\in\mathbb{Z}^3} c[\mathbf{n}]\beta^m\big(\mathbf{n}_{0;\mathbf{k}}(\mathbf{a}) + n\mathbf{n_k}(\mathbf{a}) - \mathbf{n}\big). \qquad (8)$$

### 3. OPTIMIZATION

### 3.1. *Cost function*

Given the volume with position and orientation $\mathbf{a}$, the cost function measures the dissimilarity between the $q$th C-arm image $p_q$ and the $q$th volume projection $p_q(\mathbf{a})$ at $Q$ viewing angles (*i.e.*, $q = 1,2,\ldots,Q$). We first subtract the mean intensity from a C-arm image, and then divide by its standard deviation to obtain an intensity range that depends only weakly on the power of the illumination source. We do the same for the corresponding projection. Thus normalized, the C-arm

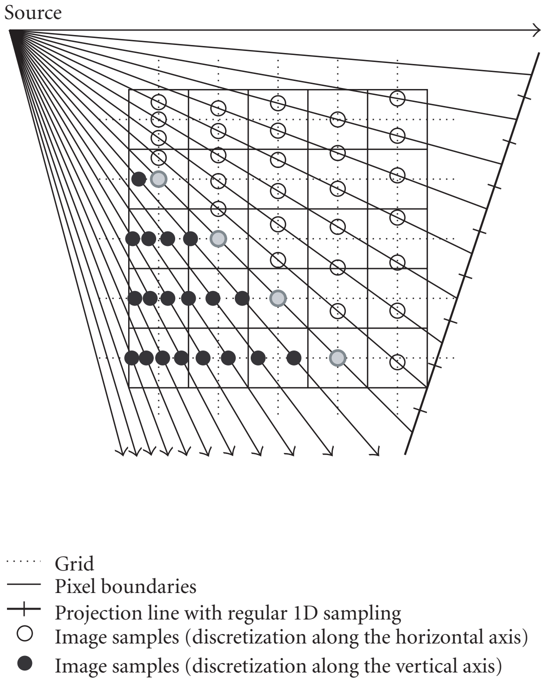

Figure 4: Shearing approach to perform the cone-beam projection of a 2D image onto a 1D line.

image and its corresponding projection can then be compared by the mean-square cost function given by

$$J(\mathbf{a}) = \frac{1}{2}\sum_{q=1}^{Q}\frac{1}{\operatorname{card}(\mathbb{D}_q)}\sum_{\mathbf{k}\in\mathbb{D}_q}\left(\frac{p_{q;\mathbf{k}}(\mathbf{a})-P_q(\mathbf{a})}{\sigma_q(\mathbf{a})}-\frac{p_{q;\mathbf{k}}-P_q}{\sigma_q}\right)^2, \tag{9}$$

where $P_q(\mathbf{a})$ and $\sigma_q(\mathbf{a})$ are the mean value and the standard deviation of the $q$th projection, respectively, and where $P_q$ and $\sigma_q$ are the mean value and the standard deviation of the $q$th C-arm image, respectively. The arbitrary binary mask $\mathbb{D}_q$ contains $\operatorname{card}(\mathbb{D}_q)$ pixels. It is suited to the $q$th view and prevents the cost function to be evaluated over irrelevant data such as the surgical instruments, the dynamic reference base, the markers if any, or nonanatomical data. For the experiments performed here, we have created this mask manually. However, for practical applications, it could also have been created using a semiautomated or perhaps automatic method.

### 3.2. Optimizer

Suited to least-squares cost functions, the LM algorithm achieves a good tradeoff between the robust but generally inefficient method of steepest descent and the efficient but nonrobust Newton method [36]. It uses the gradient

$$[\nabla J(\mathbf{a})]_i = \frac{\partial J(\mathbf{a})}{\partial a_i}, \quad i = 1, 2, \ldots, 6, \tag{10}$$

and the Hessian

$$[\nabla^2 J(\mathbf{a})]_{i,j} = \frac{\partial^2 J(\mathbf{a})}{(\partial a_i\,\partial a_j)}, \quad i, j \in \{1, 2, \ldots, 6\}. \tag{11}$$

Let $\mathbf{H}$ be a modified Hessian such that the diagonal elements of the true Hessian are multiplied by a factor $(1+\lambda)$ while its off-diagonal elements are not changed,

$$[\mathbf{H}(\mathbf{a})]_{i,j} = [\nabla^2 J(\mathbf{a})]_{i,j}(1+\lambda\delta_{i,j}), \tag{12}$$

where $\delta_{i,j} = 1 - |\operatorname{sign}(i-j)|$ is the Kronecker symbol, with $i, j \in \{1, 2, \ldots, 6\}$. Then, the LM optimization algorithm can be described by

$$\mathbf{a}^{(k+1)} = \mathbf{a}^{(k)} - \left(\mathbf{H}\left(\mathbf{a}^{(k)}\right)\right)^{-1}\nabla J\left(\mathbf{a}^{(k)}\right). \tag{13}$$

Equation (13) approximates the gradient algorithm for $\lambda \to +\infty$, albeit with vanishing steps. Similarly, it approximates the Newton algorithm for $\lambda \to 0$. The parameter $\lambda$ is adaptively tuned to provide a smooth transition from the steepest-descent algorithm (used in the beginning) to the Newton algorithm (used as approaching to the solution) [36].

When using a specific approximation of the Hessian, the LM algorithm has even better convergence properties. We describe this approximation below.

### 3.3. Gauss-Newton approximation of the Hessian

The exact components of the Hessian matrix depend on the first derivatives of the volume projections with respect to the parameter $\mathbf{a}$, as well as on their second derivatives. Since the influence of the second-derivative terms can be destabilizing when the current guess is far from the solution, we use an approximation of the elements of the Hessian matrix which ignores them [36]. This approximation makes the optimization work better because it provides a Hessian matrix that is positive-definite when $\lambda$ is sufficiently large. This is known as the Gauss-Newton approximation of the Hessian for least-squares cost functions [37, 38]. We give a detailed description of this approximation in Appendix C.

### 3.4. Gradient

The gradient of the cost function depends on the first derivatives of the volume projections with respect to the parameter $\mathbf{a}$. In turn, these derivatives depend on the spatial gradient of the volume. The spatial gradient of our B-spline data model (1) is given by

$$\nabla f(\mathbf{x}) = \sum_{\mathbf{n}\in\mathbb{Z}^N} c[\mathbf{n}]\nabla\beta^m(\mathbf{x}-\mathbf{n}). \tag{14}$$

For the B-spline of degree $m \geq 2$, we have the guarantee that its first derivative $\mathrm{d}\beta^m(x)/\mathrm{d}x$ is continuous, which is not the case if $m = 0$ (nearest-neighbor interpolation) or $m = 1$ (linear interpolation). We choose to model the data using cubic B-splines ($m = 3$), because this model provides a gradient of the cost function which is well defined everywhere. At the same time, this choice offers a good tradeoff between computational cost and interpolation quality. Detailed expressions for calculating the first derivatives of the volume projections and elements of the gradient are given in Appendix B.

### 4. EXPERIMENTS

### 4.1. Methodology

We show an application of our algorithm on the registration of a 3D CT of a frozen human spine with respect to a set of its C-arm images. We compare the computed alignment with the alignment that we estimate based on a set of markers implanted on the specimen before the data acquisition. The true alignment is therefore known *a priori*, albeit within some nonzero tolerance. To simulate clinical conditions, we register one vertebra at a time, which severely limits the amount of available data. However, to get an upper bound to the accuracy of our algorithm when registering this data set, we also perform a joint registration of several vertebrae. The data set is described in Section 4.2. The measure of the registration accuracy is given in Section 4.3. One of the elements that determines the working range of our algorithm is given in terms of the angle between the image planes. Given the CT volume and a pair of C-arm images, our preliminary experiments have shown that this angle should be at least 15°. Our algorithm therefore requires at least two input C-arm images.

We performed two sets of experiments. In the first set, which is presented in Section 4.4, we investigate the role of multiresolution. In the second set, which is described in Section 4.5, we show the benefit of increasing the number of views, and explore the influence of the minimal angle between views. We discuss the residual misalignment in Section 4.6.

### 4.2. Spine data

A human cadaver spine specimen was frozen so that it can be treated as a truly rigid body. This gives us the opportunity to jointly consider more data (here, three vertebrae) than is available in clinical practice where each vertebra can exhibit independent motion, thus providing an upper bound to the accuracy of the registration of this data set. However, to be closer to a clinical configuration, we restrict the focus of our experiments on a single vertebra by masking out large areas from the C-arm images so that only one vertebra shows through at any one time. Five fiducial markers (custom-made, titanium) were implanted on the spine. One was placed in the L5, two in the L4, and two in the L3 vertebra.

The specimen, placed in a plastic bag that is apparent in the transversal and sagittal views of Figure 5, was CT-scanned (GE LightSpeed Ultra CT scanner) with seventy two slices of size (512 × 512), in pixel units. The intraslice pixel size was (0.36 × 0.36), in mm units, and the interslice thickness was 2.5 mm. The tilt angle was zero.

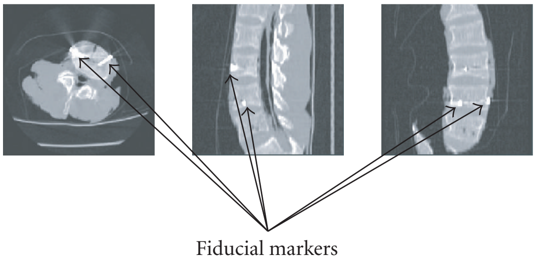

Figure 5: 3D CT with visible fiducial markers transversal (left), sagittal (center), and frontal (right) CT slices.

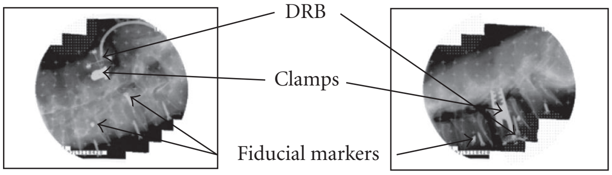

Figure 6: Two C-arm images with fiducial markers and the dynamic reference base (DRB). A superimposed grid of crosses was used to unwarp the images in a calibration step.

The images were acquired using a Siemens ISO-C C-arm instrumented with LED markers. Their positions were tracked using an optoelectronic position sensor (Northern Digital Optotrak 3020). The geometric parameters of the C-arm X-ray projection (the position and the orientation of the image plane and the position of the illumination source **s**) were determined according to the two-step procedure proposed by Hofstetter et al. [7]. The captured images were un-distorted before further use [7]. We used an optical tracking system and calibration procedure that resulted in the navigation error being 0.5 ± 0.5 mm [7].

Typical clinical settings involve a device called a dynamic reference base (DRB) to define the patient coordinate system (P-COS) in which the tools are tracked. In spinal surgery, the DRB is commonly clamped to one of the vertebrae; both DRB and clamp are therefore visible in the C-arm images. Their presence challenges the registration algorithm because neither DRB nor clamp is present in the CT data. Thanks to the mask $\mathbb{D}_q$ in (9), we have hidden them, which further reduces the amount of data available for registration; in addition, we have hidden the fiducial markers from the C-arm images to forfeit any illegitimate help from them. Two out of the seven available images are shown in Figure 6. The images were of size (768 × 576), in pixel units, and the pixel size was (0.36 × 0.36), in mm units. We show in Figure 7 the projections of the CT that correspond to the C-arm images of Figure 6, as found by our registration algorithm.

To determine the ground-truth registration, the coordinates of the fiducial markers were digitized in the P-COS using an optoelectronically tracked pointer. Then, they were transformed to the R-COS. The coordinates of the fiducial markers in the V-COS were estimated by fitting a sphere to the marker heads.

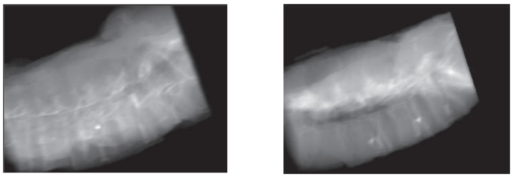

(a) (b)

Figure 7: Two projections of the CT volume. According to the outcome of our registration procedure, these projections correspond to the C-arm images given in Figure 6. As the DRB was not present at the time the CT was acquired, it is not present here. The fiducial markers, however, are present at all times.

Table 1: Volume (CT) and image (C-arm) pyramid size in voxel/pixel units.

| Level | CT | C-arm |
|---|---|---|
| 3 | $64 \times 64 \times 72$ | $96 \times 72$ |
| 2 | $128 \times 128 \times 72$ | $192 \times 144$ |
| 1 | $256 \times 256 \times 72$ | $384 \times 288$ |
| 0 | $512 \times 512 \times 72$ | $768 \times 576$ |

Table 2: Accuracy of the registration of the CT volume to eighteen pairs of C-arm images, at each level $K$ of a multiresolution pyramid ($K = \infty$: initial condition). The angle between image planes is larger than 15°. The results for a single vertebra can be compared to those for joint vertebrae (L3/L4/L5).

| $K$ | Accuracy (mm) | | | |
|---|---|---|---|---|
| | L3 | L4 | L5 | L3/L4/L5 |
| $\infty$ | 9.0 | 9.0 | 9.0 | 9.0 |
| 3 | $3.1 \pm 0.7$ | $3.5 \pm 2.6$ | $2.0 \pm 0.6$ | $1.8 \pm 0.5$ |
| 2 | $2.5 \pm 0.4$ | $3.3 \pm 3.4$ | $1.9 \pm 0.4$ | $1.7 \pm 0.5$ |
| 1 | $2.4 \pm 0.5$ | $3.3 \pm 3.4$ | $2.0 \pm 0.4$ | N/A |
| 0 | $2.4 \pm 0.5$ | $3.2 \pm 3.4$ | $2.0 \pm 0.4$ | N/A |

Table 3: Noncumulative time spent at each level $K$ of a multiresolution pyramid for the registration of the CT volume to pairs, triplets, and quadruplets of C-arm images of a single vertebra, in seconds on a Pentium III 700 MHz.

| $K$ | Time (s) | | |
|---|---|---|---|
| | 2 views | 3 views | 4 views |
| 3 | $17 \pm 4$ | $27 \pm 7$ | $37 \pm 9$ |
| 2 | $70 \pm 27$ | $102 \pm 43$ | $139 \pm 65$ |
| 1 | $411 \pm 113$ | $571 \pm 164$ | $718 \pm 233$ |
| 0 | $748 \pm 253$ | $1161 \pm 377$ | $1423 \pm 472$ |

### 4.3. Measure of the registration accuracy

We transform the V-COS coordinate $\mathbf{n}$ of each CT voxel into the R-COS by using two transformations: the ground-truth transformation $\mathbf{B}$ and the transformation $\mathbf{B}(\mathbf{a})$ estimated by our algorithm. We define the misregistration as the average of the norm of the difference between the two R-COS coordinates over all the CT voxels,

$$\varpi = \frac{1}{\mathrm{card}(\mathbb{F})} \sum_{\mathbf{n} \in \mathbb{F}} \left|\left|(\mathbf{B} - \mathbf{B}(\mathbf{a}))\mathbf{n}\right|\right|, \qquad (15)$$

where $\mathbb{F}$ is the support of $f$. In the case of perfect registration, which is achieved when $\mathbf{B}(\mathbf{a}) = \mathbf{B}$, we have that $\varpi = 0$.

To measure the registration accuracy, we evaluate (15) for $\mathbf{B}$ that is estimated using a given list of coordinates of the fiducial markers in the R-COS ($\mathbf{v}_i$, $i = 1, \dots, 5$) and in the V-COS ($\mathbf{n}_i$, $i = 1, \dots, 5$). The estimation of $\mathbf{B}$ is done by minimizing $\chi^2 = (1/5) \sum_{i=1}^{5} \|\mathbf{v}_i - \mathbf{B}\mathbf{n}_i\|^2$ in terms of $\mathbf{B}$.

We compute a single ground truth for both single-vertebra and several-vertebrae registration. More precisely, we use the collection of all five markers to determine it, even though some are not placed on the vertebra of interest in the single-vertebra case.

### 4.4. Multiresolution

We use four-level CT-volume and C-arm-image pyramids (Table 1). These pyramids are dyadic in only two directions; we do not change the number of CT slices while performing the data reduction since the registration fails otherwise, the initial number of 72 CT slices becoming too small when reduced. For the experiment performed in this section, we created a set of eighteen pairs of C-arm images such that the angle between their planes was larger than 15°.

We run the algorithm for an imposed deliberate initial misregistration such that $\varpi = 9.0$. For each vertebra, we report in Table 2 how the registration accuracy evolve across the levels of the multiresolution pyramid. In the same table, we present the results of the registration based on all three vertebrae. In the single-vertebra case, one can note that pyramid levels 0 and 1 (finest and next-to-finest levels, resp.) contribute to an accuracy gain lesser than 0.2 mm. Since this does not justify the huge additional registration time, we stopped the registration process in the several-vertebrae case after having completed the two coarsest pyramid levels.

As expected, the data reduction from three vertebrae to a single one results in a drop of performance. This effect is particularly strong for the vertebra L4 (the fourth vertebra in the lumbar part of the vertebral column) which suffers from a very small ROI obtained after having masked out the DRB and the clamps. We will see in Section 4.5 that this drop can be compensated by increasing the number of imaging views at the input and the minimal angle between the image planes.

We report in the first column of Table 3 the time spent at the $K$th level of this multiresolution pyramid. We observe that the progression is not a geometric one: while the noncumulative registration time spent until convergence at level $K = 2$ is about four times longer than that at level $K = 3$, this convergence time is nearly six times longer at $K = 1$ than at $K = 2$. The largest computational payoff, due to the

Table 4: Accuracy of the registration of the CT volume to pairs, triplets, and quadruplets of single-vertebra C-arm images for different minimal angles between image planes. The pooled performance is reported under the heading ⟨L⟩. The results for single vertebrae can be compared to those for joint vertebrae (L3/L4/L5).

| Angle | Views | Cases | Accuracy (mm) | | | | |
|---|---|---|---|---|---|---|---|
| | | | L3 | L4 | L5 | ⟨L⟩ | L3/L4/L5 |
| 15° | 2 | 18 | 2.4 ± 0.5 | 3.2 ± 3.4 | 2.0 ± 0.4 | 2.5 ± 2.0 | 1.7 ± 0.5 |
| 15° | 3 | 22 | 2.1 ± 0.2 | 1.7 ± 0.4 | 1.8 ± 0.2 | 1.9 ± 0.3 | 1.4 ± 0.2 |
| 15° | 4 | 13 | 2.0 ± 0.1 | 1.5 ± 0.3 | 1.7 ± 0.1 | 1.7 ± 0.3 | 1.4 ± 0.1 |
| 25° | 2 | 15 | 2.2 ± 0.3 | 1.8 ± 0.4 | 1.9 ± 0.3 | 2.0 ± 0.3 | N/A |
| 25° | 3 | 13 | 2.0 ± 0.1 | 1.5 ± 0.4 | 1.7 ± 0.1 | 1.7 ± 0.3 | N/A |
| 25° | 4 | 4 | 1.9 ± 0.1 | 1.5 ± 0.2 | 1.7 ± 0.1 | 1.7 ± 0.2 | N/A |

multiresolution strategy, comes between $K = 0$ and $K = 1$ since at level $K = 0$ the algorithm spent only twice the time spent at level $K = 1$, despite the fact that there is four times more data to process at $K = 0$. This suggests that the solution reached at the next-to-finest resolution is already very close to the solution found at the finest resolution, an interpretation that is consistent with the results of Table 2.

### 4.5. Several-views experiments and angle between views

We repeated the single-vertebra experiment described in Section 4.4 on two additional image sets: a set of twenty two image triplets and a set of thirteen image quadruplets, the angle between any two image planes in each set being larger than 15°. Also, we performed the same experiment using three more sets: fifteen pairs, thirteen triplets, and four quadruplets of C-arm images for the minimal angle between image planes of 25°.

We show the results in Table 4, where the influence of the number of views is combined with that of the minimal angle between views. In the same table, we present the results of the joint registration of all three vertebrae based on two, three, and four image views, and the minimal angle between image planes of 15°. We observe a significant gain in performance when using three views instead of two, while the gain of an additional fourth view, although less impressive, is present too.

We also observe that it is beneficial to enforce that the angle between views is closer to being perpendicular. For two views, a spectacular effect of increasing the minimal admissible angle from 15° to 25° is a strong reduction in the standard deviation of the registration accuracy. We interpret this reduction as indicative of the disappearance of outliers. Finally, we observe that the overall geometrical performance is similar when using four views that meet at an angle that is allowed to become rather flat, or when using only three views that meet at a less acute angle. The latter case is more favorable because of a smaller radiation dosage and because of the registration speed (Table 3). Note that if we stop the registration at level $K = 2$, as suggested in Section 4.4, the registration of the CT to three C-arm images takes just about two minutes.

### 4.6. Residual misalignment

As we can see from Table 4, our algorithm registers our CT/C-arm data set with an accuracy of about 1.7 ± 0.5 mm when using pairs of images that encompass all three vertebrae and that meet at an angle larger than 15°. But three views are needed to reach the same accuracy when only a single vertebra is available; moreover, the imaging planes should meet at an angle larger than 25° in this case. Then, our algorithm achieves an accuracy of about 1.7 ± 0.3 mm (Table 4).

The residual misalignment can be explained by the fact that some errors were made when digitizing the fiducial markers (the mean navigation error was 0.5 mm), and that some more errors were made when determining the CT indexes of the markers. The maximum error committed when determining the CT indexes of the markers was about 0.6 mm (25% slice off). This means that in the experiments carried out, our algorithm is perhaps responsible only for a fraction of the reported misregistration. Note that the mean misregistration of 1.7 mm is clearly subvoxel with respect to the interslice CT thickness of 2.5 mm.

### 5. CONCLUSION

We have developed a projection-based algorithm for the registration of a CT volume to a set of C-arm images, which takes advantage of a continuous model of the CT volume based on cubic B-splines. This model allows for a well-defined gradient of the dissimilarity measure, which is a necessary condition for efficient and accurate registration. We compute the projections of the volume using a novel method for integrating the volume along simulated rays. The advantage of this method is the replacement of a 3D interpolation by a faster 2D interpolation. Our algorithm improves robustness and speed using a coarse-to-fine registration strategy based on spline multiresolution data pyramids.

We have shown the performance of the algorithm when registering a set of human cadaver spine CT/C-arm data. Their true alignment was estimated using a set of fiducial markers implanted on the specimen before the data acquisition. We have provided an objective geometric measure of the quality of the registration.

We have found that our algorithm requires at least two input C-arm images whose planes meet at an angle larger than 15°. To register a single vertebra at a time, we performed two sets of experiments. In each set, we used image pairs, image triplets, and image quadruplets as input. In the first set, we have selected them in such a way that the minimal angle between any two image planes was 15°. In the second set, the minimal angle was chosen to be 25°. We showed that the registration accuracy could be improved by increasing the number of C-arm images and/or the minimal angle between them. Moreover, we showed that a good tradeoff between the radiation dosage, the registration accuracy, and the registration time could be achieved with three different C-arm orientations. Given the triplets of C-arm images that meet at an angle larger than 25°, our algorithm achieves registration accuracy of 1.7 ± 0.3 mm for this CT/C-arm data set. Note that this accuracy is subvoxel with respect to the CT which has an interslice thickness of 2.5 mm.

However, our algorithm was able to achieve an accuracy of 1.4 ± 0.2 mm when jointly registering all three vertebrae using triplets of images that meet at an angle larger than 15°. This shows a potential of our algorithm to register a single vertebra with smaller misalignments than 1.7 ± 0.3 mm. To achieve this, one should perhaps let the minimal angle between the image planes be closer to perpendicular. Unfortunately, the configuration of our data did not allow us to presently investigate this issue.

To be applied in a real clinical procedure, our algorithm should be validated using more realistic data such as data with soft tissues and patient data.

### APPENDICES

### A. MATRIX $\mathbf{B}(\mathbf{a})$

Let us write the vector of orientational and translatonal parameters $\mathbf{a} = (\varphi, \theta, \psi, \Delta x_1, \Delta x_2, \Delta x_3)$, where $\varphi$, $\theta$, and $\psi$ are three Euler angles, and $\Delta x_1$, $\Delta x_2$, and $\Delta x_3$ are three translations. Given $\mathbf{a}$, the voxel size $\lambda_1 \times \lambda_2 \times \lambda_3$, and the 3D index of the volume origin $\mathbf{c}$, we write the transformation $\mathbf{B}(\mathbf{a})$ as follows:

$$\mathbf{B}(\mathbf{a}) = \mathbf{T}\mathbf{\Lambda}\mathbf{C}^{-1}\mathbf{R}_1\mathbf{R}_2\mathbf{R}_3\mathbf{C}. \quad \text{(A.1)}$$

The matrix

$$\mathbf{C} = \begin{pmatrix} 1 & 0 & 0 & -[\mathbf{c}]_1 \\ 0 & 1 & 0 & -[\mathbf{c}]_2 \\ 0 & 0 & 1 & -[\mathbf{c}]_3 \\ 0 & 0 & 0 & 1 \end{pmatrix} \quad \text{(A.2)}$$

makes the origin of the volume be the center of rotation. The matrix

$$\mathbf{R}_3 = \begin{pmatrix} \cos(\psi) & -\sin(\psi) & 0 & 0 \\ \sin(\psi) & \cos(\psi) & 0 & 0 \\ 0 & 0 & 1 & 0 \\ 0 & 0 & 0 & 1 \end{pmatrix} \quad \text{(A.3)}$$

rotates the volume by the angle $\psi$ around its $z$-axis. Similarly,

$$\mathbf{R}_2 = \begin{pmatrix} \cos(\theta) & 0 & \sin(\theta) & 0 \\ 0 & 1 & 0 & 0 \\ -\sin(\theta) & 0 & \cos(\theta) & 0 \\ 0 & 0 & 0 & 1 \end{pmatrix} \quad \text{(A.4)}$$

rotates the volume by the angle $\theta$ around its new $y$-axis, while

$$\mathbf{R}_1 = \begin{pmatrix} 1 & 0 & 0 & 0 \\ 0 & \cos(\varphi) & -\sin(\varphi) & 0 \\ 0 & \sin(\varphi) & \cos(\varphi) & 0 \\ 0 & 0 & 0 & 1 \end{pmatrix} \quad \text{(A.5)}$$

rotates the volume by the angle $\varphi$ around its new $x$-axis. $\mathbf{C}^{-1}$ shifts back the index of the volume origin, and

$$\mathbf{\Lambda} = \begin{pmatrix} \lambda_1 & 0 & 0 & 0 \\ 0 & \lambda_2 & 0 & 0 \\ 0 & 0 & \lambda_3 & 0 \\ 0 & 0 & 0 & 1 \end{pmatrix} \quad \text{(A.6)}$$

scales the volume indexes by the voxel size. Finally,

$$\mathbf{T} = \begin{pmatrix} 1 & 0 & 0 & \Delta x_1 \\ 0 & 1 & 0 & \Delta x_2 \\ 0 & 0 & 1 & \Delta x_3 \\ 0 & 0 & 0 & 1 \end{pmatrix} \quad \text{(A.7)}$$

translates the volume by $\Delta x_1$, $\Delta x_2$, and $\Delta x_3$ along its new $x$-, $y$-, and $z$-axes, respectively.

The sense of rotation for $\varphi$, $\theta$, and $\psi$ has been chosen so as to follow the conventions of a right-handed coordinate system.

### B. GRADIENT OF THE COST FUNCTION

The gradient of the cost function (9) with respect to some parameter $a_i$ is given by

$$\frac{\partial J(\mathbf{a})}{\partial a_i} = \sum_{q=1}^{Q} \frac{1}{\operatorname{card}(\mathbb{D}_q)} \times \sum_{\mathbf{k}\in\mathbb{D}_q} \left( -\frac{1}{\sigma_q^2(\mathbf{a})} \frac{\partial \sigma_q(\mathbf{a})}{\partial a_i} \left(p_{q;\mathbf{k}}(\mathbf{a}) - P_q(\mathbf{a})\right) + \frac{1}{\sigma_q(\mathbf{a})} \left( \frac{\partial p_{q;\mathbf{k}}(\mathbf{a})}{\partial a_i} - \frac{\partial P_q(\mathbf{a})}{\partial a_i} \right) \right) e_{q;\mathbf{k}}(\mathbf{a}), \quad \text{(B.1)}$$

where

$$e_{q;\mathbf{k}}(\mathbf{a}) = \frac{p_{q;\mathbf{k}}(\mathbf{a}) - P_q(\mathbf{a})}{\sigma_q(\mathbf{a})} - \frac{p_{q;\mathbf{k}} - P_q}{\sigma_q}. \quad \text{(B.2)}$$

Because variations tend to average themselves over the whole image, we do not expect the mean value $P_q(\mathbf{a})$ and the standard deviation $\sigma_q(\mathbf{a})$ to exhibit a strong dependence on a small variation of $a_i$. By contrast, we consider as relevant the local dependence of the projection $p_{q;\mathbf{k}}(\mathbf{a})$. Thus, we simplify (B.1) into

$$\frac{\partial J(\mathbf{a})}{\partial a_i} \approx \sum_{q=1}^{Q} \frac{1}{\operatorname{card}(\mathbb{D}_q)} \frac{1}{\sigma_q(\mathbf{a})} \sum_{\mathbf{k}\in\mathbb{D}_q} \frac{\partial p_{q;\mathbf{k}}(\mathbf{a})}{\partial a_i} e_{q;\mathbf{k}}(\mathbf{a}). \quad (B.3)$$

From (7), we write that the unexplained term in (B.3) is

$$\begin{aligned}\frac{\partial p(\mathbf{a})}{\partial a_i} &= \frac{\partial \lambda(\mathbf{a})}{\partial a_i} \sum_{n\in\mathbb{Z}} f(\mathbf{x}_n(\mathbf{a})) \\ &\quad + \lambda(\mathbf{a}) \sum_{n\in\mathbb{Z}} \left(\nabla f(\mathbf{x}_n(\mathbf{a}))\right)^{\top} \frac{\partial \mathbf{x}_n(\mathbf{a})}{\partial a_i},\end{aligned} \quad (B.4)$$

where, for the sake of brevity, we have dropped the dependence on $q$ and $\mathbf{k}$, and where the spatial gradient of the image $\nabla f(\mathbf{x}_n(\mathbf{a}))$, evaluated at $\mathbf{x}_n(\mathbf{a}) = \mathbf{n}_0(\mathbf{a}) + n\mathbf{n}(\mathbf{a})$, is determined by (14). Here, we leave as an exercise to the reader the task to develop further the term $\partial\lambda(\mathbf{a})/\partial a_i$, which depends on geometry alone; the same goes for the term $\partial\mathbf{x}_n(\mathbf{a})/\partial a_i$.

Combining (B.4) with (1), (2), and (14), we finally get

$$\begin{aligned}\frac{\partial p}{\partial a} &= \frac{\partial \lambda}{\partial a} \sum_{n\in\mathbb{Z}} \sum_{\mathbf{n}\in\mathbb{Z}^3} c[\mathbf{n}] \prod_{i=1}^{3} \beta^3\left([\mathbf{x}_n]_i - n_i\right) \\ &\quad + \lambda \sum_{n\in\mathbb{Z}} \sum_{j=1}^{3} \frac{\partial f(\mathbf{x}_n)}{\partial x_j} \frac{\partial [\mathbf{x}_n]_j}{\partial a}\end{aligned} \quad (B.5)$$

with

$$\frac{\partial f(\mathbf{x}_n)}{\partial x_j} = \sum_{\mathbf{n}\in\mathbb{Z}^3} c[\mathbf{n}] \dot{\beta}^3\left([\mathbf{x}_n]_j - n_j\right) \prod_{i=1, i\neq j}^{3} \beta^3\left([\mathbf{x}_n]_i - n_i\right) \quad (B.6)$$

and [29]

$$\dot{\beta}^3(x) = \beta^2\left(x + \frac{1}{2}\right) - \beta^2\left(x - \frac{1}{2}\right). \quad (B.7)$$

### C. SIMPLIFIED HESSIAN

Taking an additional derivative of (B.3) results in

$$\begin{aligned}\frac{\partial^2 J(\mathbf{a})}{\partial a_i\, \partial a_j} &\approx \sum_{q=1}^{Q} \frac{1}{\operatorname{card}(\mathbb{D}_q)} \frac{1}{\sigma_q(\mathbf{a})} \sum_{\mathbf{k}\in\mathbb{D}_q} \frac{\partial p_{q;\mathbf{k}}(\mathbf{a})}{\partial a_i} \frac{\partial p_{q;\mathbf{k}}(\mathbf{a})}{\partial a_j} \frac{1}{\sigma_q(\mathbf{a})} \\ &\quad + \sum_{q=1}^{Q} \frac{1}{\operatorname{card}(\mathbb{D}_q)} \frac{1}{\sigma_q(\mathbf{a})} \sum_{\mathbf{k}\in\mathbb{D}_q} \frac{\partial^2 p_{q;\mathbf{k}}(\mathbf{a})}{\partial a_i \partial a_j} e_{q;\mathbf{k}}(\mathbf{a}),\end{aligned} \quad (C.1)$$

where, as before, we have ignored the terms $\partial\sigma/\partial\mathbf{a}$ and $\partial P/\partial\mathbf{a}$. As explained in Section 3.3, it is actually *beneficial* to also ignore the second-derivative terms. Finally, we express a component of the Hessian as

$$\left[\nabla^2 J(\mathbf{a})\right]_{i,j} \approx \sum_{q=1}^{Q} \frac{1}{\operatorname{card}(\mathbb{D}_q)} \frac{1}{\sigma_q^2(\mathbf{a})} \sum_{\mathbf{k}\in\mathbb{D}_q} \frac{\partial p_{q;\mathbf{k}}(\mathbf{a})}{\partial a_i} \frac{\partial p_{q;\mathbf{k}}(\mathbf{a})}{\partial a_j}, \quad (C.2)$$

where the remaining differential terms are available from (B.5).

### ACKNOWLEDGMENTS

This work was supported in part by Grant 02-U61 from the AO ASIF Foundation, Switzerland, and in part by the Swiss National Science Foundation under Grant 2153-066938.01/1. We thank Dr. Carlos Óscar Sánchez Sorzano from the Escuela Politécnica Superior, Universidad San Pablo-CEU, Madrid, Spain. His suggestions helped in enhancing the quality of the manuscript.

### REFERENCES

[1] E. Bainville, I. Bricault, P. Cinquin, and S. Lavallée, “Concepts and methods of registration for computer-integrated surgery,” in *Computer Assisted Orthopedic Surgery (CAOS)*, L.-P. Nolte and R. Ganz, Eds., pp. 15–34, Hogrefe & Huber, Seattle, Wash, USA, 1999.

[2] P. J. Edwards, D. J. Hawkes, G. P. Penney, and M. J. Clarkson, “Guiding therapeutic procedures,” in *Medical Image Registration*, J. V. Hajnal, D. L. G. Hill, and D. J. Hawkes, Eds., pp. 253–278, CRC Press LLC, Boca Raton, Fla, USA, 2001.

[3] B. Fei, J. L. Duerk, D. T. Boll, J. S. Lewin, and D. L. Wilson, “Slice-to-volume registration and its potential application to interventional MRI-guided radio-frequency thermal ablation of prostate cancer,” *IEEE Transactions on Medical Imaging*, vol. 22, no. 4, pp. 515–525, 2003.

[4] N. Sugano, “Computer-assisted orthopedic surgery,” *Journal of Orthopaedic Science*, vol. 8, no. 3, pp. 442–448, 2003.

[5] U. Berlemann, M. Slomczykowski, F. Langlotz, et al., “Computerassistenz in der Orthopädischen Chirurgie—Grundlegende Konzepte und Weiterentwicklungen durch Integration der Fluoroskopie,” *Sportorthopädie-Sporttraumatologie*, vol. 15, no. 3, pp. 155–159, 1999.

[6] L.-P. Nolte, M. A. Slomczykowski, U. Berlemann, et al., “A new approach to computer-aided spine surgery: fluoroscopy-based surgical navigation,” *European Spine Journal*, vol. 9, pp. S78–S88, 2000.

[7] R. Hofstetter, M. Slomczykowski, M. Sati, and L.-P. Nolte, “Fluoroscopy as an imaging means for computer-assisted surgical navigation,” *Computer Aided Surgery*, vol. 4, no. 2, pp. 65–76, 1999.

[8] W. Birkfellner, J. Wirth, W. Burgstaller, et al., “A faster method for 3D/2D medical image registration—a simulation study,” *Physics in Medicine and Biology*, vol. 48, no. 16, pp. 2665–2679, 2003.

[9] Á. Czopf, C. Brack, M. Roth, and A. Schweikard, “3D-2D registration of curved objects,” *Periodica Polytechnica Series—Electrical Engineering*, vol. 43, no. 1, pp. 19–41, 1999.

[10] J. Feldmar, N. Ayache, and F. Betting, “3D-2D projective registration of free-form curves and surfaces,” *Computer Vision and Image Understanding*, vol. 65, no. 3, pp. 403–424, 1997.

[11] A. Guéziec, P. Kazanzides, B. Williamson, and R. H. Taylor, “Anatomy-based registration of CT-scan and intraoperative X ray images for guiding a surgical robot,” *IEEE Transactions on Medical Imaging*, vol. 17, no. 5, pp. 715–728, 1998.

[12] A. Hamadeh, P. Sautot, S. Lavallée, and P. Cinquin, “Towards automatic registration between CT and X-ray images: cooperation between 3D/2D registration and 2D edge detection,” in *Proceedings of International Symposium on Medical Robotics and Computer Assisted Surgery (MRCAS ’95)*, pp. 39–46, Baltimore, Md, USA, November 1995.

[13] D. LaRose, J. Bayouth, and T. Kanade, “Transgraph: interactive intensity-based 2D/3D registration of X-ray and CT data,” in

*Medical Imaging 2000: Image Processing*, K. M. Hanson, Ed., vol. 3979 of *Proceedings of SPIE*, pp. 385–396, San Diego, Calif, USA, February 2000.

[14] S. Lavallée and R. Szeliski, "Recovering the position and orientation of free-form objects from image contours using 3D distance maps," *IEEE Transactions on Pattern Analysis and Machine Intelligence*, vol. 17, no. 4, pp. 378–390, 1995.

[15] H. Livyatan, Z. Yaniv, and L. Joskowicz, "Gradient-based 2 D/3-D rigid registration of fluoroscopic X-ray to CT," *IEEE Transactions on Medical Imaging*, vol. 22, no. 11, pp. 1395–1406, 2003.

[16] T. Rohlfing, D. B. Russakoff, J. Denzler, K. Mori, and C. R. Maurer Jr., "Progressive attenuation fields: fast 2D-3D image registration without precomputation," *Medical Physics*, vol. 32, no. 9, pp. 2870–2880, 2005.

[17] A. Hamadeh and P. Cinquin, "Kinematic study of lumbar spine using functional radiographies and 3D/2D registration," in *Proceedings of 1st Joint Conference, Computer Vision, Virtual Reality and Robotics in Medicine and Medical Robotics and Computer-Assisted Surgery (CVRMed-MRCAS '97)*, J. Troccaz, E. Grimson, and R. Mosges, Eds., pp. 109–118, Grenoble, France, March 1997.

[18] D. Sarrut and S. Clippe, "Geometrical transformation approximation for 2D/3D intensity-based registration of portal images and CT scan," in *Proceedings of the 4th International Conference on Medical Image Computing and Computer-Assisted Intervention (MICCAI '01)*, W. Niessen and M. Viergever, Eds., pp. 532–540, Utrecht, The Netherlands, October 2001.

[19] L. M. Gottesfeld Brown and T. E. Boult, "Registration of planar film radiographs with computed tomography," in *IEEE Workshop on Mathematical Methods in Biomedical Image Analysis (MMBIA '96)*, pp. 42–51, San Francisco, Calif, USA, June 1996.

[20] L. Lemieux, R. Jagoe, D. R. Fish, N. D. Kitchen, and D. G. T. Thomas, "A patient-to-computed-tomography image registration method based on digitally reconstructed radiographs," *Medical Physics*, vol. 21, no. 11, pp. 1749–1760, 1994.

[21] G. P. Penney, J. Weese, J. A. Little, P. Desmedt, D. L. G. Hill, and D. J. Hawkes, "A comparison of similarity measures for use in 2-D-3-D medical image registration," *IEEE Transactions on Medical Imaging*, vol. 17, no. 4, pp. 586–595, 1998.

[22] D. B. Russakoff, T. Rohlfing, K. Mori, et al., "Fast generation of digitally reconstructed radiographs using attenuation fields with application to 2D-3D image registration," *IEEE Transactions on Medical Imaging*, vol. 24, no. 11, pp. 1441–1454, 2005.

[23] J. Weese, R. Göcke, G. P. Penney, P. Desmedt, T. M. Buzug, and H. Schumann, "Fast voxel-based 2D/3D registration algorithm using a volume rendering method based on the shear-warp factorization," in *Image Processing*, vol. 3661 of *Proceedings of SPIE*, pp. 802–810, San Diego, Calif, USA, February 1999.

[24] P. Thévenaz, U. E. Ruttimann, and M. Unser, "A pyramid approach to subpixel registration based on intensity," *IEEE Transactions on Image Processing*, vol. 7, no. 1, pp. 27–41, 1998.

[25] P. Thévenaz and M. Unser, "Optimization of mutual information for multiresolution image registration," *IEEE Transactions on Image Processing*, vol. 9, no. 12, pp. 2083–2099, 2000.

[26] A. Aldroubi, M. Unser, and M. Eden, "Cardinal spline filters: stability and convergence to the ideal sinc interpolator," *Signal Processing*, vol. 28, no. 2, pp. 127–138, 1992.

[27] I. J. Schoenberg, "Contributions to the problem of approximation of equidistant data by analytic functions," *Quarterly of Applied Mathematics*, vol. 4, no. 2, pp. 45–99, 112–141, 1946.

[28] P. Thévenaz, T. Blu, and M. Unser, "Interpolation revisited," *IEEE Transactions on Medical Imaging*, vol. 19, no. 7, pp. 739–758, 2000.

[29] M. Unser, "Splines: a perfect fit for signal and image processing," *IEEE Signal Processing Magazine*, vol. 16, no. 6, pp. 22–38, 1999.

[30] P. Brigger, F. Müller, K. Illgner, and M. Unser, "Centered pyramids," *IEEE Transactions on Image Processing*, vol. 8, no. 9, pp. 1254–1264, 1999.

[31] M. Unser, A. Aldroubi, and M. Eden, "The $L_2$ polynomial spline pyramid," *IEEE Transactions on Pattern Analysis and Machine Intelligence*, vol. 15, no. 4, pp. 364–379, 1993.

[32] M. Unser, A. Aldroubi, and M. Eden, "B-spline signal processing. II. Efficiency design and applications," *IEEE Transactions on Signal Processing*, vol. 41, no. 2, pp. 834–848, 1993.

[33] T. Blu, P. Thévenaz, and M. Unser, "MOMS: maximal-order interpolation of minimal support," *IEEE Transactions on Image Processing*, vol. 10, no. 7, pp. 1069–1080, 2001.

[34] A. W. Paeth, "A fast algorithm for general raster rotation," in *Proceedings of Graphics Interface '86—Vision Interface '86*, pp. 77–81, Vancouver, BC, Canada, May 1986.

[35] A. Tanaka, M. Kameyama, S. Kazama, and O. Watanabe, "A rotation method for raster image using skew transformation," in *Proceedings of IEEE Computer Society Conference on Computer Vision and Pattern Recognition (CVPR '86)*, pp. 272–277, Miami Beach, Fla, USA, June 1986.

[36] W. H. Press, B. P. Flannery, S. A. Teukolsky, and W. T. Vetterling, *Numerical Recipes: The Art of Scientific Computing*, Cambridge University Press, Cambridge, UK, 1988.

[37] D. P. Bertsekas, *Nonlinear Programming*, Athena Scientific, Belmont, Mass, USA, 1999.

[38] C. T. Kelley, *Iterative Methods for Optimization*, SIAM, Philadelphia, Pa, USA, 1999.

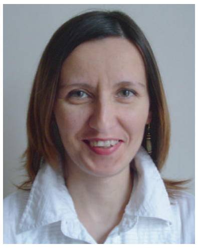

**S. Jonić** was born in 1970 in Negotin, Serbia and Montenegro. She obtained the B.S. and M.S. degrees from the Faculty of Electrical Engineering, Belgrade, Serbia and Montenegro, in 1996 and 1999, respectively. In 1999, she joined the Biomedical Imaging Group at the École Polytechnique Fédérale de Lausanne (EPFL), Lausanne, Switzerland, where she received a Ph.D. degree in 2003. Since 2004, she is with the University Pierre and Marie Curie, Paris, France. Her research interests include image processing techniques for image-guided surgery and for three-dimensional electron microscopy.

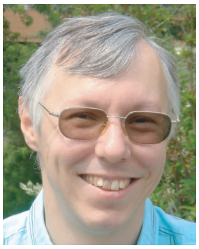

**P. Thévenaz** was born in 1962 in Lausanne, Switzerland. He graduated from the École Polytechnique Fédérale de Lausanne (EPFL) in 1986. He then joined the Institute of Microtechnology of the University of Neuchâtel, Switzerland, where he obtained a Ph.D. degree in 1993. From 1994 to 1996, he was a Visiting Fellow with the Biomedical Engineering and Instrumentation Program (BEIP), National Center for Research Resources (NCRR), National Institutes of Health (NIH), Bethesda, Md, USA, where he developed research interests that include splines and multiresolution signal representations, geometric image transformations, visualization, and biomedical image registration. In 1996, he became a Visiting Associate. From 1998 to 2001, he is with the École Polytechnique Fédérale de Lausanne (EPFL), in the Biomedical Imaging Group of the Institute of Imaging and Applied Optics. Since 2004, he is the Associate Editor for the IEEE Transactions on Medical Imaging.

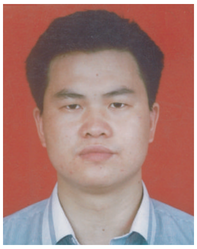

**G. Zheng** graduated from the First Military Medical University (now renamed as Southern Medical University), China, in 1992 with a B.S. degree, and in 1995 with an M.S. degree, both in biomedical engineering. He earned his Ph.D. in biomedical engineering from University of Bern in 2002. He is currently a Postdoctoral Fellow and a Group Leader of the same university at the Institute for Surgical Technology and Biomechanics, MEM Research Center for Orthopaedic Surgery. His research interests include medical image processing and analysis, statistical shape reconstruction, rigid and nonrigid registrations, endoscopy-based tracking, and computer-assisted interventions with a focus on orthopaedics. He is a Member of the IEEE, the MICCAI Society, and the SPIE.

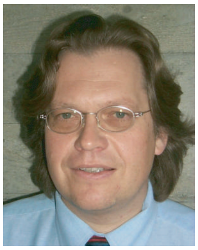

**L.-P. Nolte** is Professor for surgical technology and biomechanics at the University of Bern, Switzerland. He received the M.S. and Ph.D. degrees from the Ruhr-University Bochum, Germany, in 1980 and 1983. From 1984 to 1987, he headed the Nonlinear Shell Research Group at the Institute of Mechanics of the Ruhr-University Bochum, working in the field of elastic and elastoplastic deformations of thin-walled engineering structures. From 1987 to 1990, he established the Orthopaedic Research Group at the Ruhr-University Bochum focussing his work on spinal biomechanics. In 1990, he joined the Bioengineering Center at Wayne State University in Detroit, Mich, USA, as an Associate Professor of mechanical engineering. In collaboration with the Department of Neurosurgery, he extended the scope of his research to computer-aided surgery. In 1993, he took over the Orthopaedic Biomechanics Division at the MEM Institute for Biomechanics in Bern. Since 2001, he is the Codirector of the Swiss National Center for Competence in Research "Computer Aided and Image Guided Medical Interventions" (http://co-me.ch/) located at the Eidgerössische Technische Hochschule Zürich (ETHZ). In 2002, he became the Codirector of the MEM Research Center for Orthopaedic Surgery and the Director of the Institute for Surgical Technology and Biomechanics at the Medical Faculty of the University of Bern.

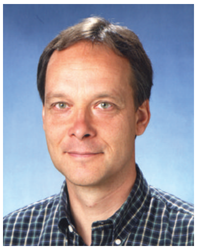

**M. Unser** received the M.S. (summa cum laude) and Ph.D. degrees in electrical engineering in 1981 and 1984, respectively, from the École Polytechnique Fédérale de Lausanne (EPFL), Switzerland. From 1985 to 1997, he worked as a Scientist with the National Institutes of Health, Bethesda, USA. He is now Professor and Director of the Biomedical Imaging Group at the EPFL. His main research area is biomedical image processing. He has a strong interest in sampling theories, multiresolution algorithms, wavelets, the use of splines for image processing, and he is the author of over 120 published journal papers in these areas. He is the Associate Editor-in-Chief of the IEEE Transactions on Medical Imaging and the Editor-in-Chief of the Wavelet Digest, the electronic newsletter of the wavelet community. He was General Chair for the first IEEE International Symposium on Biomedical Imaging (ISBI'2002), which was held in Washington, DC, July 7–10, 2002. He also chairs the newly created technical committee of the IEEE-SP Society on Bio Imaging and Signal Processing (BISP). He is a Fellow of the IEEE. He received the 1995 and 2003 Best Paper Awards and the 2000 Magazine Award from the IEEE Signal Processing Society.

